# Efficacy of l‐Arginine treatment in patients with HTLV‐1‐associated neurological disease

**DOI:** 10.1002/acn3.51715

**Published:** 2022-12-22

**Authors:** Satoshi Nozuma, Eiji Matsuura, Yuichi Tashiro, Ryusei Nagata, Masahiro Ando, Yu Hiramatsu, Yujiro Higuchi, Yusuke Sakiyama, Akihiro Hashiguchi, Kumiko Michizono, Keiko Higashi, Toshio Matsuzaki, Daisuke Kodama, Masakazu Tanaka, Yoshihisa Yamano, Takashi Moritoyo, Ryuji Kubota, Hiroshi Takashima

**Affiliations:** ^1^ Department of Neurology and Geriatrics Kagoshima University Graduate School of Medical and Dental Sciences Kagoshima Japan; ^2^ Division of Neuroimmunology, Joint Research Center for Human Retrovirus Infection Kagoshima University Kagoshima Japan; ^3^ Division of Neurology, Department of Internal Medicine St. Marianna University School of Medicine Kawasaki Japan; ^4^ Clinical Research Promotion Center The University of Tokyo Hospital Bunkyo‐ku Japan

## Abstract

**Objective:**

HTLV‐1 infection causes HTLV‐1‐associated myelopathy/tropical spastic paraparesis (HAM/TSP), resulting in loss of motor function. In this Phase 2 trial, we assessed the efficacy and safety of l‐arginine in patients with HAM/TSP.

**Methods:**

This open‐label, single‐arm, Phase 2 study enrolled patients diagnosed with HAM/TSP. Patients received l‐arginine at a dose of 20 g orally for 1 week and were followed‐up for 3 weeks. The primary endpoint was change in walking speed in the 10‐m walk test (10MWT). The main secondary endpoints were change in Timed Up and Go Test (TUGT) time, improvement in inflammatory markers in cerebrospinal fluid (CSF), safety, and tolerability.

**Results:**

The study enrolled 20 patients (13 [65%] female) with a mean age of 67.8 years (95% CI 62.3 to 73.3). Although the primary endpoint, the changes in 10MWT time between baseline (Day 0) and Day 7, did not reach statistical significance (mean percent change in time −3.5%, 95% CI −10.8% to 3.7%; *P* = 0.32), a significant improvement was detected between baseline and Day 14 (−9.4%, 95% CI −16.6% to −2.2%; *P* = 0.01). Significant improvements were also observed in selected secondary endpoints, including in TUGT time (−9.1%, 95% CI −15.5% to −2.7%; *P* < 0.01), and in neopterin concentration in CSF (−2.1 pmol/mL, 95% CI −3.8 to −0.5; *P* = 0.01). Adverse events were infrequent, mild, and resolved rapidly.

**Interpretation:**

l‐arginine therapy improved motor function and decreased CSF inflammatory markers. l‐arginine thus represents a promising therapeutic option for patients with HAM/TSP.

**Trial Registration Number:**

UMIN000023854.

## Introduction

The retrovirus human T‐lymphotropic virus type 1 (HTLV‐1) is the etiologic agent for adult T cell leukemia/lymphoma^1^ and HTLV‐1‐associated myelopathy/tropical spastic paraparesis (HAM/TSP).^2^, ^3^ HAM/TSP is clinically characterized by progressive spastic paraparesis, urinary dysfunction, and sensory impairment. Chronic spinal cord inflammation induced by HTLV‐1‐infected T cells is thought to be the cause of neural damage and degeneration in patients with HAM/TSP.^4^, ^5^ Although HAM/TSP is rarely fatal, the disease severely impacts activities of daily living^6^, ^7^ and current therapies are unsatisfactory. Most available treatments focus on suppressing the immune response or reducing HTLV‐1 proviral load (PVL); however, these treatments do not improve neurological deficits or quality of life.^8^, ^9^ Thus, there is a need to identify novel treatments that improve neurological inflammation and functional outcomes for patients with HAM/TSP.

l‐Arginine is a basic amino acid and has significant metabolic roles including a precursor for the synthesis of urea, ornithine, citrulline, and nitric oxide. Recent studies have reported that l‐arginine and its metabolism would be a potential therapeutic target in a number of diseases.^10^, ^11^ Indeed, l‐arginine is currently in clinical use for the treatment of urea cycle defects^12^ and mitochondrial diseases.^13^ Furthermore, l‐arginine has been shown to have a protective effect against the accumulation of misfolded proteins in neurons^14^, ^15^ and regulate immune responses and inflammatory reactions.^16^, ^17^ Since l‐arginine is known to cross the blood–brain barrier and has a neuroprotective effect in neurological disorders,^11^, ^18^ application of l‐arginine could be considered as a candidate therapy for HAM/TSP.

In this open‐label, single‐arm, Phase 2 trial, we evaluated the efficacy and safety of short‐term treatment with orally administered l‐arginine in patients with HAM/TSP.

## Methods

### Study design

This was an open‐label, single‐center, Phase 2 study. Patients aged ≥20 years who were diagnosed with HAM/TSP according to the World Health Organization guidelines^19^ were enrolled. Among the inclusion criteria were: treatment without immunosuppressants or with oral prednisolone therapy at a fixed dose of <10 mg daily for at least 3 months, and the capability of walking at least 10 m with or without a walking aid. Exclusion criteria were: complications of spinal cord compression such as cervical spine disease, disc herniation, ossification of the yellow ligament; psychiatric disorder, epilepsy, dementia, Parkinson's disease; dose adjustment of drugs related to treatment for HAM/TSP within the preceding 12 weeks; history of cancer; or complicated with cancer. Patients received l‐arginine at a dose of 20 g orally three times (TID) daily for 1 week and were followed‐up for 3 weeks. Full inclusion and exclusion criteria are detailed in the protocol in Supplement 1. Two board‐certified neurologists evaluated the motor function of all patients and were blind to the timing of the treatments.

### Standard protocol approvals, registrations, and patient consents

The study protocol was reviewed and approved by the Institutional Review Board of Kagoshima University. The clinical trial was registered with University Hospital Medical Information Network Clinical Trials Registry (UMIN trial number, UMIN000023854). All patients gave written informed consent prior to enrollment.

### Outcomes and disease evaluation

The primary endpoint was change in walking speed (i.e. time taken) in a 10‐m walk test (10MWT) between baseline (Day 0) to Day 7. The secondary efficacy endpoints were change in walking speed in a 10MWT from baseline to Days 3 and 14; change in Timed Up and Go Test (TUGT) time from baseline to Days 3, 7, and 14; and change in cell counts, total protein level, and neopterin concentration in the cerebrospinal fluid (CSF) from baseline to Day 7. The safety of l‐arginine was assessed throughout the study based on adverse events, vital signs, and laboratory tests.

Lower limb spasticity was investigated using the Modified Ashworth Scale^20^ and motor impairment was assessed using the Osame's Motor Disability Score (OMDS).^21^ Dysuria was evaluated using the Overactive Bladder Symptom Score (OABSS).^22^ Blood tests included the plasma l‐arginine concentration, complete blood cell count, HTLV‐1 proviral load (PVL) in peripheral blood mononuclear cells (PBMCs), and levels of biochemical markers, cytokines, and amino acids. CSF tests included cell counts and concentrations of neopterin and C‐X‐C motif chemokine ligand 10 (CXCL10), which are specific central nervous system (CNS) inflammatory markers in patients with HAM/TSP.^23^ Further details are provided in the protocol in Supplement 1.

### Statistical analyses

Percent changes in 10MWT, TUGT, and PBMC PVL; mean change in OABSS score; and mean change in serum concentrations of l‐arginine, adiponectin, ornithine, citrulline, and tryptophan were analyzed by one‐way repeated measures analysis of variance followed by Dunnett's multiple comparison test. HTLV‐1 PVL and concentrations of neopterin and CXCL10 in CSF were analyzed by paired *t*‐tests. All statistical analyses were performed using Prism version 9.3.1 (GraphPad Software). *P* < 0.05 was considered statistically significant. Values are presented as the mean and 95% confidence intervals (CI).

## Results

### Patients

A total of 20 patients with HAM/TSP who satisfied the inclusion and exclusion criteria were enrolled in the study. The patient characteristics at baseline are summarized in Table 1. The mean age of the study population was 67.8 years (95% CI 62.3 to 73.3) and the number of females and males was 13 and seven, respectively. The disease duration was 16.8 years (95% CI 11.1 to 22.5), OMDS was 4.5 (95% CI 4.0 to 4.9), and PVL in PBMCs was 814.3 copies/10^4^ cells (95% CI 406.3 to 1222.0) (Table 1).

**Table 1:** Characteristics of HAM/TSP patients at the baseline of l‐arginine trial.

Patient No.	Age	Sex	Disease duration (years)^1^	OMDS	Proviral load, copies/10^4^ PBMCs
1	81	M	18	6	3556
2	52	F	14	4	1893
3	87	F	3	4	168
4	70	M	26	2	878
5	78	M	6	5	1166
6	80	M	6	4	1290
7	58	F	23	5	73
8	71	F	33	4	173
9	60	M	17	4	474
10	64	M	44	4	698
11	69	F	5	5	99
12	41	F	6	4	90
13	65	F	14	4	282
14	83	F	33	6	125
15	57	F	27	4	1484
16	66	M	31	6	74
17	69	F	4	4	851
18	78	F	7	5	240
19	54	F	14	5	1677
20	73	F	5	4	995

OMDS, Osame Motor Disability Score.

^1^ The disease duration is calculated from the time point of the first disease symptom.

### Efficacy

**Figure 1:**
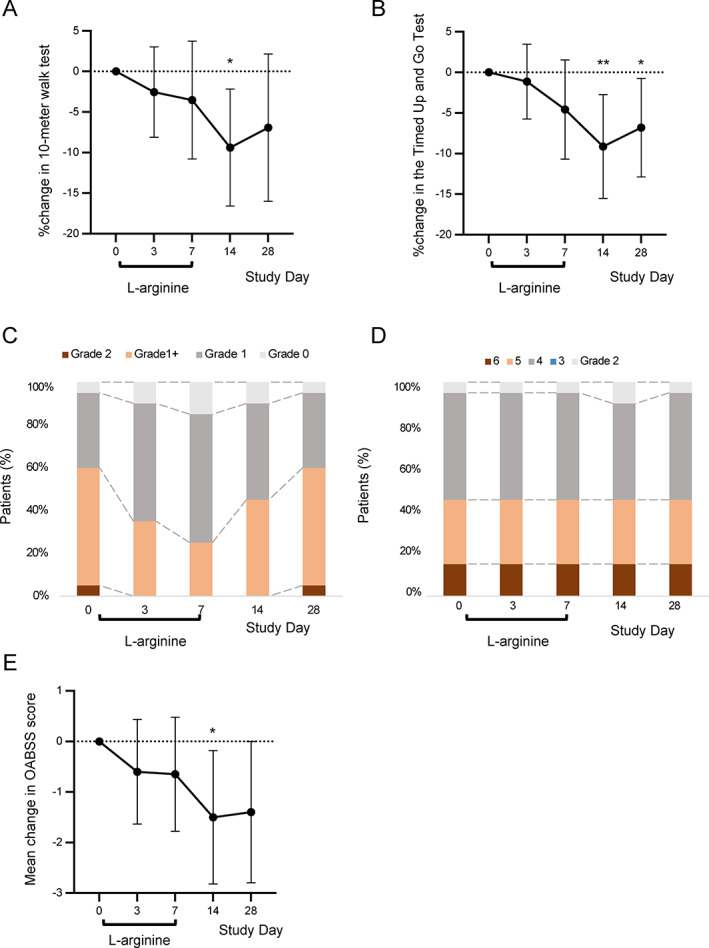
Effects of l‐arginine on motor and bladder function in patients with HAM/TSP. (A and B) Percent change from baseline in the 10‐m walk test time (A) and in the Timed Up and Go Test time (B). (C and D) Distribution of patients according to grades of the Modified Ashworth Scale (C) and Osame's Motor Disability Score (D). (E) Mean change from baseline in the Overactive Bladder Symptom Score (OABSS). Dashed bar indicates the baseline value. Black circles and vertical bars indicate mean and 95% confidence interval, respectively (A, B, and E). N = 20. *P < 0.05, **P < 0.01.

All 20 patients were included in the efficacy evaluation. Although change in walking speed in the 10MWT between baseline and Day 7, the primary endpoint, did not reach statistical significance, there was a trend towards a decrease in time taken (mean percent change −3.5%, 95% CI −10.8% to 3.7%; *P* = 0.32, Fig. 1A). Moreover, the decrease between baseline and Day 14 was significant (−9.4%; 95% CI −16.6% to −2.2%; *P* = 0.01). A significant improvement in the TUGT measure (secondary endpoint) was also observed between baseline and Day 14 (−9.1%, 95% CI −15.5% to −2.7%; *P* < 0.01) and Day 28 (−6.8%, 95% CI −12.9% to −0.8%; *P* = 0.03, Fig. 1B). These results indicate that oral administration of 20 g l‐arginine TID for 7 days improves the walking speed and balance of patients with HAM/TSP.

l‐Arginine treatment markedly improved lower limb spasticity, as measured with the Modified Ashworth Scale. The percentage of patients with grade 1+ or higher decreased from 60% at baseline to 25% at Day 7 (Fig. 1C), the final day of l‐arginine treatment. Thereafter, the lower limb spasticity gradually returned and was at baseline levels by Day 28. Overall motor disability, evaluated using OMDS, did not change during the 7‐day treatment or 3‐week observational follow‐up period (Fig. 1D).

Urinary disturbance was also significantly improved after l‐arginine administration. The OABSS score decreased by 1.5 points between baseline and Day 14 (95% CI −2.8% to −0.2%; *P* = 0.03) and by 1.4 points between baseline and Day 28 (95% CI −2.8% to 0.0%; *P* = 0.05, Fig. 1E). Thus, l‐arginine treatment ameliorated bladder dysfunction.

### Immunologic responses

**Figure 2:**
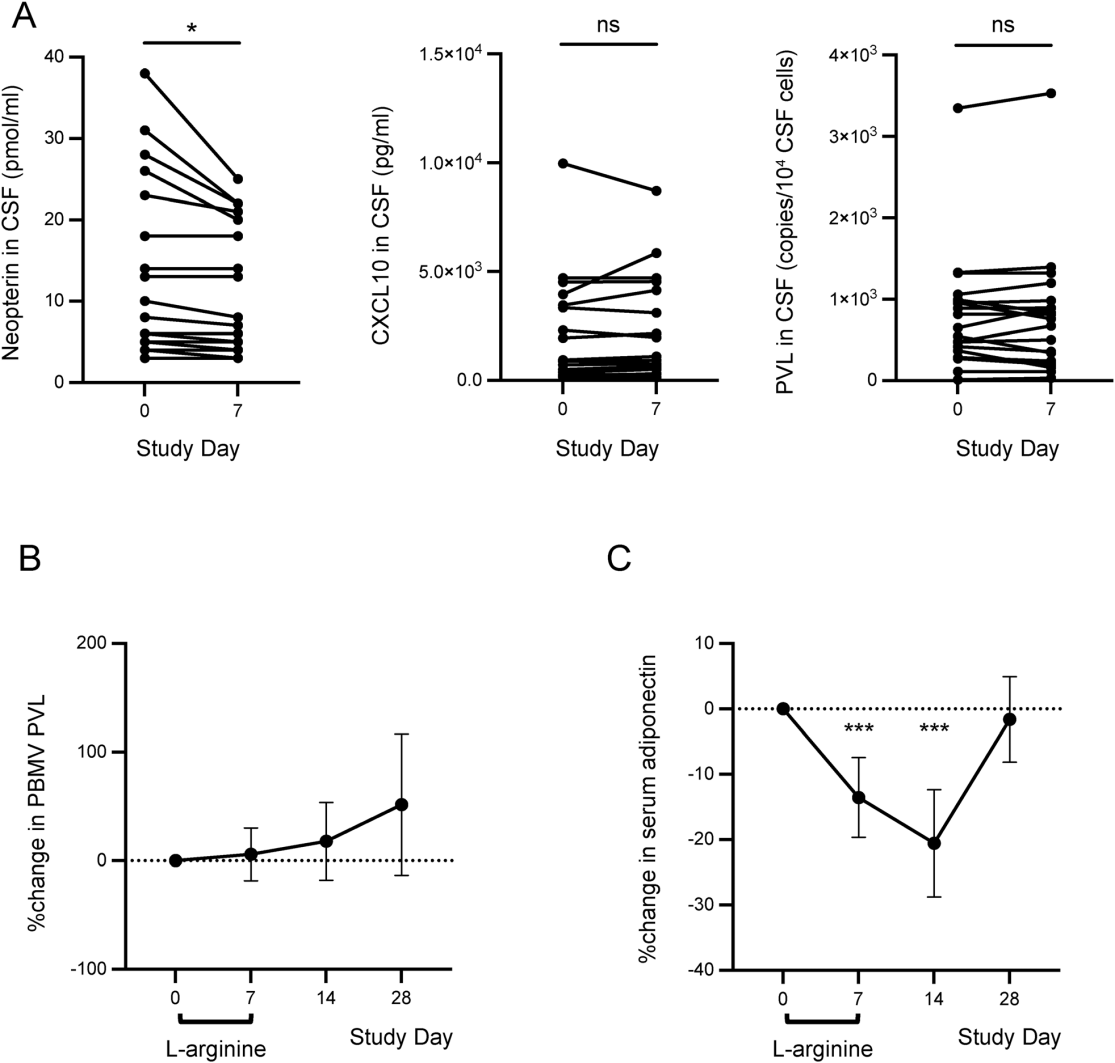
Effects of l‐arginine on immunological markers in the cerebrospinal fluid and peripheral blood of patients with HAM/TSP. (A) Concentrations of neopterin (left), CXCL10 (center), and HLTV‐1 proviral load (PVL, right) in the cerebrospinal fluid (CSF). (B and C) Percent change from baseline in peripheral blood mononuclear cell (PBMC) PVL (B) and serum adiponectin concentration (C). Dashed bar indicates the baseline value. Black circles and vertical bars indicate mean and 95% confidence interval, respectively (B and C). N = 20. *P < 0.05, ***P < 0.001.

l‐Arginine is known to cross the blood–brain barrier and is considered to have neuroprotective effects in various neurological disorders.^11^, ^18^ To examine whether l‐arginine therapy influences immune response markers in the CNS of patients with HAM/TSP, we compared the levels of various inflammatory markers in the CSF before and after l‐arginine treatment. Among the markers measured, neopterin concentrations (secondary endpoint) were decreased from 12.7 pmol/mL (95% CI 7.6 to 17.8) at baseline to 10.6 pmol/mL (95% CI 6.8 to 14.3) on Day 7, representing a significant change (−2.1 pmol/mL, 95% CI −3.8 to −0.5; *P* = 0.01, Fig. 2A). None of the other inflammatory markers measured in CSF, including total cell count, total protein concentration, PVL, or CXCL10 were significantly changed after l‐arginine treatment (Fig. 2A and Fig. S1).

No significant change in PBMC PVL was detected during the treatment or follow‐up periods (Fig. 2B). Among the serum inflammatory markers analyzed, only adiponectin showed a significant change (Fig. 2C). Serum adiponectin levels were reduced by 13.5% (95% CI −19.6% to −7.5%; *P* < 0.01) between baseline and Day 7 and by 20.6% (95% CI −28.8% to −12.4%; *P* < 0.01) between baseline and Day 14. Other serum inflammatory markers measured, including tumor necrosis factor‐α (TNF‐α) and natural killer cell counts, were not significantly changed from baseline at any time point analyzed (Fig. S1). Because the level of neopterin in the CSF is considered to be associated with disease activity in patients with HAM/TSP,^24^ these results suggest that l‐arginine may have immunomodulatory properties in this neuroinflammatory disease.

### l‐Arginine pharmacokinetics and effects on plasma amino acid profile

**Figure 3:**
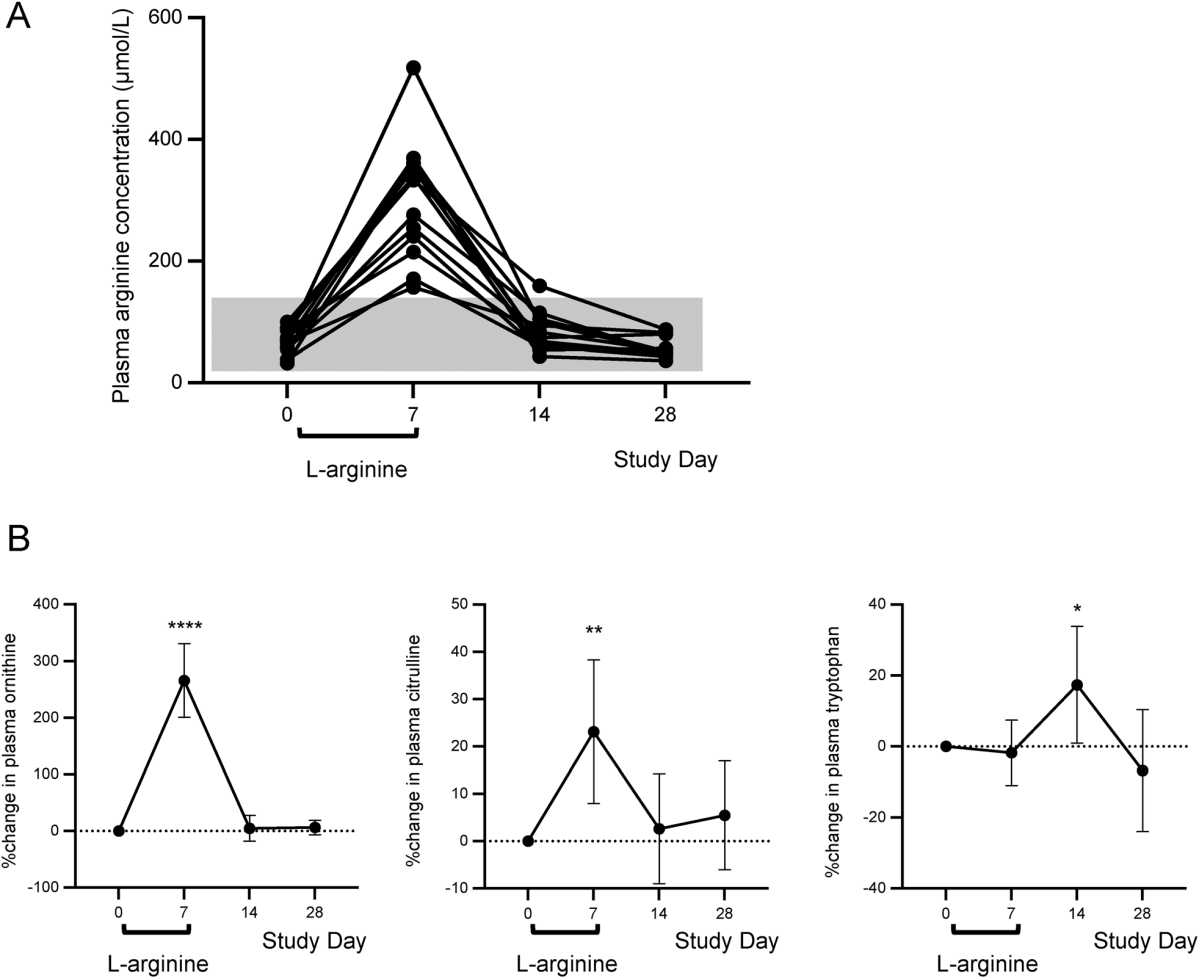
Plasma amino acid concentrations in l‐arginine‐treated patients with HAM/TSP. (A) Plasma l‐arginine concentrations of individual patients. Gray box indicates the normal plasma l‐arginine concentration range. (B) Percent change from baseline in plasma concentrations of ornithine (left), citrulline (center), and tryptophan (right). Dashed bar indicates the baseline value. Black circles and vertical bars indicate mean and 95% confidence interval, respectively (B). N = 20. *P < 0.05, **P < 0.01, ****P < 0.0001.

As expected, the mean plasma l‐arginine concentration increased markedly between baseline (68.1 μmol/L [95% CI 56.0 to 80.1; range, 32.1 to 99.5 μmol/L]) and Day 7, the end of the dosing period (303.2 μmol/L [95% CI 244.0 to 362.3; range, 156.5 to 517.6 μmol/L]). Thereafter, plasma l‐arginine levels decreased and returned to baseline levels (31.8–149.5 μmol/L by Day 28) (Fig. 3A). Thus, plasma concentrations of l‐arginine peaked on the final day of dosing and returned to normal levels by Day 28, indicating that the compound did not accumulate.

We also evaluated the effect of high doses of l‐arginine on the plasma profiles of other amino acids and identified significant changes in the plasma concentrations of three amino acids; ornithine, citrulline, and tryptophan (Fig. 3B). These results were not surprising, given that l‐arginine is converted to ornithine by arginase and then to citrulline by ornithine transcarbamylase in the urea cycle. Among the other amino acids evaluated, 10 amino acids exhibited a decrease in plasma concentration and 17 did not exhibit a concentration change over the 28‐day study period (Fig. S2). These results indicate that high doses of l‐arginine could influence the metabolism of other amino acids but the plasma concentrations had returned to baseline levels within 3 weeks of the cessation of l‐arginine administration.

### Safety

l‐Arginine treatment was well tolerated and all 20 patients completed the trial. Adverse events that occurred in at least 4 of 20 patients are listed in Table 2. Mild symptomatic events occurred in a few patients, including two patients with diarrhea and one patient with nausea at the beginning of l‐arginine treatment, but the events resolved within a few days. Laboratory abnormalities after l‐arginine treatment were increased levels of blood urea nitrogen and chloride, however, the levels were returned to the normal range on Day 7. Transient elevation of C‐reactive protein was observed in five patients and improved without any treatment. Other abnormalities were also resolved within 1 week after the cessation of l‐arginine (Table 2).

**Table 2:** Adverse events in l‐arginine trial.

Adverse event	Frequency, *n* (%)
*Symptomatic event*	
Diarrhea	2 (10)
Nausea	1 (5)
*Hematologic event*	
Blood urea nitrogen	17 (85)
Chloride	9 (45)
Blood sugar	7 (35)
Total protein	5 (25)
C‐reactive protein	5 (25)
Erythrocyte sedimentation rate	5 (25)
Red blood cell	4 (20)
Alanine aminotransferase	4 (20)
Sodium	4 (20)
Albumin	4 (20)

## Discussion

In this open‐label trial, we assessed the efficacy and safety of l‐arginine therapy in patients with HAM/TSP. Significant improvements were observed in the 10MWT at Day 14 and the TUGT at Days 14 and 28, indicating that short‐term treatment with l‐arginine has a beneficial effect on motor function in these patients. In addition, a significant reduction in neopterin concentration in the CSF was detected, suggesting that l‐arginine therapy might modulate the inflammatory status of the CNS.

HAM/TSP is a progressive neurological disorder characterized by spastic paraparesis and abnormal gait, and some patients may progress to wheelchair dependency or even become bedridden. Because HAM/TSP seriously influences activities of daily living,^6^, ^7^ there is an unmet medical need for therapeutic agents capable of slowing or improving the motor disability that profoundly impairs normal functioning. Most agents tested clinical trials in HAM/TSP have aimed to reduce the HTLV‐1 PVL and suppress the immune response, with the goal of risk reduction or clinical improvement. Currently available drugs, including corticosteroids and interferon‐α, have only a small beneficial effect on motor impairment, and the treatments are difficult to tolerate due to adverse effects.^8^, ^9^ A monoclonal antibody against C‐C chemokine receptor type 4 (CCR4), which targets HTLV‐1‐infected cells, recently failed to show effectiveness in improving motor function in HAM/TSP patients, although transient reductions in HTLV‐1 PVL and inflammatory markers were demonstrated.^25^ Therefore, the present study aimed to determine whether l‐arginine might have beneficial effects on the CNS and improves the gait disturbance and other disabilities of HAM/TSP patients without intolerable or serious adverse effects.

l‐Arginine is an endogenous basic amino acid that plays a variety of roles in metabolism, including as a precursor for the synthesis of urea, ornithine, citrulline, and nitric oxide. l‐Arginine is widely used as a supplement, and high‐dose l‐arginine therapy is in clinical use for urea cycle defects,^12^ mitochondrial myopathy, encephalopathy, lactic acidosis and stroke‐like episodes.^13^ l‐Arginine is known to cross the blood–brain barrier and is considered to be neuroprotective in various disorders, including neuroinflammatory and neurodegenerative diseases.^11^, ^18^ Recent studies have shown that l‐arginine can prevent the toxic aggregation and accumulation of amyloid β 1–42 peptide^14^ and polyglutamine protein^15^ in neurons, which contribute to the pathogenesis of Alzheimer's disease and polyglutamine diseases, including Huntington disease and various spinocerebellar ataxias, respectively. l‐Arginine administration has also been shown to ameliorate neurological symptoms and protein aggregation in animal models of neurodegenerative disorders,^15^ and several studies have shown that l‐arginine decreases levels of inflammatory cytokines such as TNF‐α and interleukin‐6 and protects neuronal cells against high glucose‐induced damage.^16^, ^17^ In the present study, we found that neopterin concentrations in the CSF were significantly decreased after l‐arginine administration. Neopterin is produced by activated monocytes/macrophages and is associated with immune cell activation^26^, ^27^; therefore, our results suggest that l‐arginine could be a modulator of inflammation in the CNS. This effect might at least partly explain the observed improvements in gait disturbance after l‐arginine administration in patients with HAM/TSP.

Spastic paraparesis in HAM/TSP is thought to be caused by chronic inflammation and diffuse degeneration of the spinal cord.^4^ However, immunohistochemical analysis of skeletal muscle tissue have shown that inflammatory and noninflammatory myopathic changes occur in more than 50% of HAM/TSP patients,^28^, ^29^ and some patients with axial myopathy involving paraspinal atrophy have been described.^30^ These observations indicate that skeletal muscle involvement is another important contributor to the motor disability in HAM/TSP patients. Notably, l‐arginine has been shown to protect against muscle damage. l‐Arginine administration to healthy volunteers increased muscle strength and improved recovery after physical exercise through increases in nitric oxide production, which promotes vasodilation in the active muscle.^31^, ^32^ Indeed, we have shown that l‐arginine improves muscle symptoms in patients with mitochondrial myopathy.^33^ In the present study, we observed that l‐arginine administration ameliorated both the neurological and muscular symptoms in patients with HAM/TSP, as evidenced by significant improvements in walking speed and gait functions. Several previous studies indicated that the l‐arginine‐nitric oxide pathway might have some side effects among neurological conditions.^11^, ^34^ Importantly, no major adverse events and laboratory abnormalities were observed during the l‐arginine treatment in our study. The mild gastrointestinal symptoms were transient and resolved within a few days of onset. The elevation of chloride was considered to be due to the drug of l‐arginine containing hydrochloride.

## Limitations

This study had several limitations. First, the sample size was small and the study lacked a control group. Given that patients generally have high anticipation of treatment efficacy in clinical trials, inclusion of a placebo group may have influenced our findings. Second, the treatment and observational periods for evaluating the efficacy and safety of l‐arginine were short. Because high doses of l‐arginine might affect amino acid and protein metabolism, the long‐term efficacy and safety of high‐dose l‐arginine needs to be clarified. Third, this study included only Japanese patients, which may be considered a geographical limitation. Finally, the study included only patients who were able to walk with or without a walking aid because 10MWT and TUGT were used for the assessment of motor function.

## Conclusions

This Phase 2 clinical trial demonstrated that oral administration of l‐arginine improved the motor function of HAM/TSP patients with minimal adverse effects. A significant decrease in neopterin levels in CSF suggest that l‐arginine might act as an immunomodulator in neuroinflammatory disorders. Overall, this study identifies oral l‐arginine administration as a promising treatment option for patients with HAM/TSP. Our study was limited by the small sample size and short duration of treatment. A larger scale placebo‐controlled study will therefore be needed to provide more robust evidence for the efficacy and safety of l‐arginine in patients with HAM/TSP with moderate disability.

## Author Contributions

E. Matsuura had full access to all of the data in the study and takes responsibility for the integrity of the data and the accuracy of the data analysis. Study concept and design: E. Matsuura, Y. Tashiro. Acquisition, analysis, or interpretation of data: S. Nozuma, E. Matsuura, Y. Tashiro, R. Nagata, M. Ando, Y. Hiramatsu, Y. Higuchi, Y, Sakiyama, A. Hashiguchi, K. Michizono, K. Higashi, T. Matsuzaki, D. Kodama, M. Tanaka, Y. Yamano, T. Moritoyo, R. Kubota, H. Takashima. Drafting of the manuscript: S. Nozuma, E. Matsuura, T. Moritoyo, R. Kubota, H. Takashima. Statistical analysis: S. Nozuma, E. Matsuura, Y. Tashiro, R. Kubota. Administrative, technical, or material support: S. Nozuma, E. Matsuura, Y. Tashiro, R. Nagata, M. Ando, Y. Hiramatsu, Y. Higuchi, Y. Sakiyama, A. Hashiguchi, K. Michizono, K. Higashi, T. Matsuzaki, R. Kubota, H. Takashima. Study supervision: T. Moritoyo, Kubota, H. Takashima.

## Funding Information

This work was supported by Grants‐in‐Aid for Research Activity Start‐up (20 K22879 to S. Nozuma) and Scientific Research (C) (21 K07418 to E. Matsuura) from the Japan Society for the Promotion of Science (JSPS), Japan.

## Conflict of Interest

The authors have declared that no conflicts of interest exist.

## Supporting information


Appendix S1

Figure S1

Figure S2

## Data Availability

Anonymized data not published within this article will be made available by request from any qualified investigator.
