# Supplementary material for: Efficacy of l‐Arginine treatment in patients with HTLV‐1‐associated neurological disease
Source: Ann Clin Transl Neurol. 2022 Dec 22;10(2):237–45. doi: 10.1002/acn3.51715 (PMC9930431; doi:10.1002/acn3.51715)
Supplement: Supplementary file 1 — Appendix S1 [file ACN3-10-237-s001.docx]

**Study protocol**

| Protocol title | Efficacy Study of L-arginine in Patients with HTLV-1 Associated Myelopathy |
| --- | --- |
| UMIN CTR Number | UMIN000023854 |
| Principal Investigator | Professor Hiroshi Takashima |
| Sponsor | Kagoshima University Hospital |

**List of Abbreviations**

| Abbreviation | Unabbreviated term |
| --- | --- |
| ALT | Alanine Aminotransferase |
| AST | Aspartate Aminotransferase |
| BUN | Blood Urea Nitrogen |
| CBC | Complete Blood Count |
| Cr | Creatinine |
| CRB | Certified Review Board |
| CRP | C-Reactive Protein |
| CSF | Cerebrospinal Fluid |
| CTCAE v4.0 | Common Terminology Criteria for Adverse Events v4.0 |
| CXCL10 | C-X-C motif chemokine 10 |
| FBS | Fasting Blood Sugar |
| HAM/TSP | HTLV-1 Associated Myelopathy/Tropical Spastic Paraparesis |
| Hb | Hemoglobin |
| HbA1c | Hemoglobin A1c |
| HTLV-1 | Human T-lymphotropic Virus Type 1 |
| IL-2R | Interleukin-2 Receptor |
| K | Potassium |
| MAS | Modified Ashworth Scale |
| Na | Sodium |
| OABSS | Overactive Bladder Symptom Score |
| OMDS | Osame's Motor Disability Score |
| PBMC | Peripheral Blood Mononuclear Cell |
| PLT | Platelet Count |
| RBC | Red Blood Cell |
| TP | Total Protein |
| VAS | Visual Analogue Scale |
| WBC | White Blood Cell |

Table of Contents

[1. Synopsis 5](#_Toc92888443)

[2. Background 7](#_Toc92888444)

[3. Trial Objectives 7](#_Toc92888445)

[3.1. Primary Objective 7](#_Toc92888446)

[3.2. Secondary Objectives 7](#_Toc92888447)

[4. Inclusion/Exclusion Criteria 7](#_Toc92888448)

[4.1. Eligibility Criteria 7](#_Toc92888449)

[4.2. Inclusion Criteria 8](#_Toc92888450)

[4.3. Exclusion Criteria 8](#_Toc92888451)

[4.4. Subjective Discontinuation Criteria 8](#_Toc92888452)

[5. Study Design and Plan 9](#_Toc92888453)

[5.1. Study Design 9](#_Toc92888454)

[5.2. Study Schedule 9](#_Toc92888455)

[**5.2.1.** **Screening** 9](#_Toc92888456)

[**5.2.2.** **Treatment Phase** 9](#_Toc92888457)

[5.3. Unexpected Exacerbation 9](#_Toc92888458)

[6. Treatment of Subjects 10](#_Toc92888459)

[6.1. Description of Study Drug 10](#_Toc92888460)

[6.2. Concomitant Medications 10](#_Toc92888461)

[6.3. Prohibited Medications 10](#_Toc92888462)

[6.4. Dosing Guidelines 10](#_Toc92888463)

[6.5. Informed Consent 11](#_Toc92888464)

[7. Study Drug Materials 11](#_Toc92888465)

[7.1. Study Drug 11](#_Toc92888466)

[7.2. Drug Administration 11](#_Toc92888467)

[8. Assessment of Activity 11](#_Toc92888468)

[8.1. Primary and Secondary Endpoints 11](#_Toc92888469)

[8.2. Evaluation points 12](#_Toc92888470)

[9. Assessment of Safety 15](#_Toc92888471)

[9.1. Safety evaluation indicators 15](#_Toc92888472)

[9.2. Assessment of Adverse Events 16](#_Toc92888473)

[9.3. Recording Adverse Events 16](#_Toc92888474)

[9.4. Treatment and Follow-Up of Adverse Events 16](#_Toc92888475)

[10. Statistics 16](#_Toc92888476)

[10.1. Data analysis 16](#_Toc92888477)

[10.2. Statistical Analysis Plan 16](#_Toc92888478)

[11. Access to Source Data/Documents 17](#_Toc92888479)

[12. Quality control and Quality Assurance 17](#_Toc92888480)

[12.1. Monitoring 17](#_Toc92888481)

[12.2. Audit 17](#_Toc92888482)

**List of Table**

[Table 1: Schedule of Assessments 19](#_Toc92809833)

# **Synopsis**

| Title of Study | Efficacy Study of L-arginine in Patients with HTLV-1 Associated Myelopathy |
| --- | --- |
| Primary Objective | Change in 10-meter walking time from Day 0 to Day 7 |
| Secondary Objective | - Change in 10-meter walking time from Day 0 to Days 3 and 14 - Change in measurements obtained with the Timed Up and Go Test from Day 0 to Day 3, 7, and 14 - Degree of improvement in CSF cell count, protein, and neopterin concentration - Safety; Adverse events (nature and severity of adverse events, frequency of discontinuations and dropouts) |
| Study Drug | Argi U® Combination Granules |
| Active Ingredient | L-Arginine hydrochloride and L-Arginine |
| Number of Patients | 20 patients will be recruited |
| Study Entry Criteria | HTLV-1 associated myelopathy/Tropical spastic paraparesis (HAM/TSP)  Inclusion Criteria:   1. Patient with HAM/TSP 2. Age ≥ 20 years at the time of consent 3. Ongoing medication for HAM/TSP, with no changes in 12 weeks before enrollment. Subjects on maintenance therapy with steroids must have been receiving ≤ 10 mg/day prednisolone equivalent continuously for at least 12 weeks before enrollment. 4. Subjects are able to walk ≥ 10 meters at screening (use of a single cane, double canes or any walking aid is allowed) 5. Voluntary written informed consent to participate in the study 6. Subjects are able to see the Kagoshima University Hospital on the schedule days   Exclusion Criteria:   1. Participation in a clinical trial within 16 weeks before informed consent 2. 10-meter walking time at screening has changed more than 30% compared with 10-meter walking time within 8 weeks before consent (data of all the days when visiting multiple days) 3. Any of the following significant concomitant diseases; Poorly controlled diabetes mellitus, Heart failure, Severe chronic lung disease requiring oxygen therapy, Renal failure, Liver dysfunction 4. Active malignancy or past history of malignancy (with the exception of resected or surgically cured solid cancer, with no recurrence of the cancer more than 3 years before informed consent, and resected intra-epithelium carcinoma of cervix cancer, uterine body or digestive ducts) 5. Adult T cell leukemia/lymphoma 6. Pregnant, breastfeeding or possible pregnant. Subjects without agree to contraception during the study period. 7. Concurrent spinal cord compression lesion (e.g., cervical spine disease and ossification of the yellow ligament) with the exception of conditions that would not affect evaluating 10-meter walking test in the study, as judged by the investigator 8. Concurrent diseases that markedly impair brain function such as psychiatric disorder, epilepsy, dementia, Parkinson’s disease, cerebrovascular disease 9. Bone fracture within 24 weeks before informed consent 10. Any other condition unsuitable for participation in the study in the opinion |

# **Background**

Since the retrovirus HTLV-1 cannot be eliminated from HTLV-1 carrier patients, HAM/TSP is thought to be a lifelong inflammatory condition once it develops. Therefore, therapeutic agents for HAM/TSP are required to continuously control the inflammation caused by the virus. In this respect, long-term administration of corticosteroids is reasonable, as the drug is used in some other chronic autoimmune diseases. However, the steroids show small therapeutic benefit, and continuing the treatment is difficult to tolerate due to various side effects including diabetes and osteoporosis. Since HAM/TSP requires lifelong treatment, there is a need for treatment with inexpensive and safe drugs. In this context, we have experienced cases of improvement in gait disturbance in several HAM/TSP patients treated with L-arginine. In addition, CSF neopterin level also improved in the patients. Treatment with amino acids may be an inexpensive and safe way to treat the patients with HAM/TSP. However, there is no evidence that L-arginine is effective in treating the patients, and clinical studies are needed to confirm its efficacy.

# **Trial Objectives**

There are only a few effective treatments for HAM/TSP, and we will search for a safe, inexpensive, and long-term oral treatment. We will conduct an open study in a small number of patients to confirm the efficacy and safety of Argi U® (L-arginine), which is already approved for the treatment of amino acid metabolism disorders, for patients with HAM/TSP.

## **Primary Objective**

Change in 10-meter walking time from Day 0 to Day 7.

## **Secondary Objectives**

- Efficacy
- Change in 10-meter walking time from Day 0 to Days 3 and 14.
- Change in measurements obtained with the Timed Up and Go Test from Day 0 to Day 3, 7, and 14.
- Degree of improvement in CSF cell count, protein, and neopterin concentration
- Safety
- Adverse events (nature and severity of adverse events, frequency of discontinuations and dropouts)

# **Inclusion/Exclusion Criteria**

## **Eligibility Criteria**

Target disease; HTLV-1 associated myelopathy/Tropical spastic paraparesis：HAM/TSP (Diagnostic criteria according to World Health Organization conference at Kagoshima in 1988)

## **Inclusion Criteria**

Subjects eligible for enrollment in the study must meet all of the following criteria:

1. Patient with HAM/TSP
2. Age ≥ 20 years at the time of consent
3. Ongoing medication for HAM/TSP, with no changes in 12 weeks before enrollment. Subjects on maintenance therapy with steroids must have been receiving ≤ 10 mg/day prednisolone equivalent continuously for at least 12 weeks before enrollment.
4. Subjects are able to walk ≥ 10 meters at screening (use of a single cane, double canes or any walking aid is allowed)
5. Voluntary written informed consent to participate in the study
6. Subjects are able to see the Kagoshima University Hospital on the schedule days

## **Exclusion Criteria**

1. Participation in a clinical trial within 16 weeks before informed consent
2. 10-meter walking time at screening has changed more than 30% compared with 10-meter walking time within 8 weeks before consent (data of all the days when visiting multiple days)
3. Any of the following significant concomitant diseases; Poorly controlled diabetes mellitus, Heart failure, Severe chronic lung disease requiring oxygen therapy, Renal failure, Liver dysfunction
4. Active malignancy or past history of malignancy (with the exception of resected or surgically cured solid cancer, with no recurrence of the cancer more than 3 years before informed consent, and resected intra-epithelium carcinoma of cervix cancer, uterine body or digestive ducts)
5. Adult T cell leukemia/lymphoma
6. Pregnant, breastfeeding or possible pregnant. Subjects without agree to contraception during the study period
7. Concurrent spinal cord compression lesion (e.g., cervical spine disease, ossification of the yellow ligament) with the exception of conditions that would not affect evaluating 10-meter walking test in the study, as judged by the investigator)
8. Concurrent diseases that markedly impair brain function such as psychiatric disorder, epilepsy, dementia, Parkinson’s disease, cerebrovascular disease
9. Bone fracture within 24 weeks before informed consent
10. Any other condition unsuitable for participation in the study in the opinion of the investigator

## **Subjective Discontinuation Criteria**

This clinical study will be discontinued if serious side effects beyond the expected range are observed with medication, or if it can be determined that the study drug is clearly ineffective. As this is a clinical study involving a small number of patients, data related to this study will be analyzed after discontinuation to provide basic information for clarifying the causes of adverse events.

# **Study Design and Plan**

## **Study Design**

An open interventional study of Argi U® (L-arginine) orally for 7 consecutive days to confirm safety and efficacy in a single group of HAM/TSP patients without a placebo or control group.

## **Study Schedule**

### **Screening**

The principal investigator or sub-investigator will assign a screening number (HAM-SCR01~) to each subject whose consent has been obtained, conduct a screening test to confirm eligibility, and investigate that there are no problems with inclusion in this study. The screened cases will be listed in the screening roster. The principal investigator or sub-investigator will enroll the subjects whose eligibility has been confirmed by the screening test into this study. The study phases are described below and a schedule are displayed in Table 1.

### **Treatment Phase**

- Study drug administration (Day 1)

The principal investigator or sub-investigator will confirm that there are no problems with the administration of the study drug based on the test results at the time of admission. Confirm that the disbursed drugs are taken appropriately.

- Discharge from the hospital (Day 14)

The investigator or sub-investigator will continue to evaluate the efficacy and safety of the drug after the end of the administration period.

- Termination and discontinuation (Day 28)

The principal investigator or sub-investigator will conduct an exit examination on Day 28. If events requiring follow-up or adverse events persist during the exit examination, they shall be followed until safety is confirmed.

## **Unexpected Exacerbation**

In the event of an unexpected exacerbation during this clinical study, the following treatments will be provided in consultation with the patient

- Oral or intravenous corticosteroid therapy

Adverse events: Diabetes, osteoporosis, immunosuppression, gastric and duodenal ulcer, etc.

- Interferon alpha injection therapy

Adverse events: Fever, chills, malaise, bone marrow suppression, anemia, dizziness, anorexia, hypoglycemia, interstitial pneumonia, liver dysfunction, rheumatoid arthritis, etc.

# **Treatment of Subjects**

## **Description of Study Drug**

Trade name: Argi U® Combination Granules

Composition: The drug contains the following ingredients in 1.3g

L-Arginine hydrochloride 605mg

L-Arginine 500mg

(Total 1000mg as L-Arginine)

This product is a white granule with no odor and a slightly peculiar taste.

## **Concomitant Medications**

Treatment during the clinical study will continue as before hospitalization, including rehabilitation. If symptoms worsen significantly during the clinical study, the study will be stopped immediately and treatment will be given according to the possible condition and cause. Changes in the dosage and administration of the following medications and treatments will not be permitted during study participation. Rehabilitation and massage should not be changed in frequency or duration.

- Other immunosuppressive drugs and steroids (except topical drugs)
- All drugs prescribed to improve its symptoms
- Rehabilitation and massage
- Health foods (supplements)

## **Prohibited Medications**

The following drugs are prohibited from the time of screening until the end of the study period.

- Injectable steroids (may be used at the discretion of the physician)
- Interferon
- Other study drugs

## **Dosing Guidelines**

Argi U® Combination Granules 0.5 g/kg/day should be divided into 3 or 4 doses and taken orally for 7 consecutive days. Start with a small dose, specifically half the planned daily dose on the first day. On the second day, three quarters of the planned dose should be administered, and from the third day, the planned dose of 0.5 g/kg/day should be administered. If blood samples taken after the start of administration show minor abnormalities that may be caused by the drug, or if it is difficult to take the scheduled dose due to taste or other problems, the daily dose should be reduced. In such cases, the daily dosage will remain the same. Discontinue medication if blood samples show markedly abnormal values or if symptoms of the primary disease worsen. In addition, if impairment due to acidemia occurs, administration should be discontinued immediately. Since the metabolic half-life of amino acids is remarkably fast, the possibility of prolonged disability is low, and symptoms should improve quickly. If there is no improvement in symptoms considered to be adverse effects, the best medical treatment should be provided as appropriate. After completion of the study, treatment will continue as before the study and there will be no restrictions on further medical treatment.

## **Informed Consent**

The principal investigator or sub-investigator will select candidate patients for this study, provide them with sufficient explanations using the explanation document and consent form approved by the Kagoshima University Clinical Research Review Committee, and obtain free and voluntary consent in writing from the patients themselves.

# **Study Drug Materials**

## **Study Drug**

　Trade name: Argi U® Combination Granules

　Composition: The drug contains the following ingredients in 1.3g

　L-Arginine hydrochloride 605mg

　L-Arginine 500mg

　(Total 1000mg as L-Arginine)

　This product is a white granule with no odor and a slightly peculiar taste.

　Packaging: 500g

## **Drug Administration**

The usual daily dosage is 0.15 to 0.50 g (0.12 to 0.38 g as L-arginine) per kilogram of body weight in 3 to 6 divided doses. The dosage may be adjusted according to the patient's age and symptoms. In general, start with a small dose and titrate upward until a satisfactory effect is obtained, referring to blood ammonia levels, subjective symptoms, etc.

# **Assessment of Activity**

Table 1: Schedule of Assessments

## **Primary and Secondary Endpoints**

The primary endpoint:

- Change in 10-meter walking time from Day0 to Day7

The secondary endpoints:

- Efficacy
- Change in 10m walking time between Day 0 and Days 3 and 14
- Change in measurements obtained with the Timed Up and Go from Day 0 to Day 3, 7, and 14
- Degree of improvement in CSF cell count, protein, and neopterin concentration

## **Evaluation points**

Evaluation points are described below and the schedule are displayed in Table 1.

1. Subject’s Background (Day -28)

Initials, sex, date of birth, date of initial diagnosis, date of confirmed diagnosis of HAM/TSP (year, month or year only is acceptable), medical history, treatment history, pre-existing conditions, complications, concomitant medications

1. Vital signs (Day -28, 0, 1, 3, 7, 14, 28)

Body temperature, blood pressure, pulse, height (measured only on Day-28), weight (not measured on Day 1 and 3)

1. Blood Test (Day -28, 0, 7, 14, 28)

The following items shall be measured in the hospital, and the method of measurement shall be standardized during the study period. Hematological examination (collection volume: 4ml); WBC, RBC, Hb, PLT. Blood biochemical examination (collection volume: 8ml); TP, AST, ALT, BUN, Cr, Na, K, FBS, CRP. However, anti-HTLV-1 antibody titer (CLIA method) will be measured only for Day-28 (screening), and HbA1c will be measured only for Day0 (admission). On Day0 and Day7, Day14, and Day28, HTLV-1 proviral load in PBMC (blood collection volume: 20 ml) will be measured.

The following items shall be measured in the PASOLAB, and the measurement methods shall be standardized during the research period.

Serum sIL-2R, Serum anti-HTLV-1Ab titer (CLIA) (collection volume: 10ml)

1. Blood gas analysis (conducted as necessary on Day 3) (Blood collection volume: 2 ml)

Measurement shall be performed in the hospital, and the measurement method shall be standardized during the study period.

1. Blood concentration (Day 0, 7, 14, 28)

Blood Arginine and amino acids concentration shall be measured by external testing system (BML).

1. Urinalysis (Day- 28, 0, 7, 14, 28)

Measurements shall be taken in the hospital, and the measurement method shall be standardized during the study period.

1. CSF examination (Day 0, 7) (collection volume: 5ml)

The following items shall be measured in the hospital, and the method of measurement shall be standardized during the study period.

Cell count, protein

The following items will be measured in the PASOLAB, and the measurement methods will be standardized during the study period.

CSF Anti-HTLV-1 antibody titer (CLIA method), CSF neopterin concentration, CSF CXCL10

1. 10-meter walk test (Day -28, 0, 3, 7, 14, 28)

＜Implementation Method＞

Mark the beginning and end of a 10 m straight line on the floor with tape, etc., and measure the time required for walking with a stopwatch.

If a walking aid (one-handed cane, two-handed cane, etc.) is used for walking, it will be measured without changing the aid during the study period.

The test will be conducted after a short stretching session before the walking test. (The content of stretching will be decided for each patient.)

Start walking 2 m before the starting point, and measure the time taken from the time when the patient passes the starting point to the time when the patient passes the ending point.

1. Time Up and Go Test (Day -28, 0, 3, 7, 14, 28)

＜Implementation Method＞

The starting position is leaning back lightly, with hands on thighs.

The starting posture is to lean back lightly on the backrest and place the hands on the thighs. In this case, both feet should be on the floor.

The time taken to stand up from the armchair, walk around the marker 3m away, and sit down on the chair again shall be measured. 0m point shall be the front leg of the chair, and 3m point shall be the center of the cone.

The subject is asked to perform a series of movements at "normal walking speed" and "maximum walking speed" (two times in total) following the call of the measurer.

The measurer measures the time from the time when a part of the subject's body starts to move to the time when the buttocks touch the ground. (This is not a strict rule; it should be from standing to sitting.)

After two measurements, the smaller value (faster time) shall be adopted, and the number of seconds to one decimal place shall be recorded. (The second digit shall be rounded off.)

[Precautions for measurement]

If a walking aid is used in daily life, it should be used as it is. If you use a walking aid in your daily life, use it as it is.

The way the subject turns the cone is up to the subject.

When the subject goes around the cone, there is a high risk of falling, and when the subject sits down, there is a risk of falling due to excessive momentum, so the measurer should be careful. (Be especially careful on tatami mats, as they are slippery.)

1. OMDS (Day -28, 0, 3, 7, 14, 28)

<Implementation methods>

The patient’s condition will be determined according to the table below and scored.

| Grade | Motor Disability Grade |
| --- | --- |
| 0 | Normal gait and running |
| 1 | Normal gait but runs slowly |
| 2 | Abnormal gait (staggering or spastic) |
| 3 | Unable to run |
| 4 | Need handrail to climb stairs |
| 5 | Needs a cane (unilateral support) to walk |
| 6 | Needs bilateral support to walk |
| 7 | Can walk 5-10m with bilateral support |
| 8 | Can walk 1-5m with bilateral support |
| 9 | Cannot walk, but able to crawl |
| 10 | Cannot crawl, but able to move using arms |
| 11 | Cannot move around, but able to turn over in bed |
| 12 | Cannot turn over in bed |
| 13 | Cannot even move toes |

1. Modified Ashworth Scale (Day -28, 0, 3, 7, 14, 28)

The patient’s condition will be determined according to the table below and scored.

| Grade | Description |
| --- | --- |
| 0 | No increase muscle tone |
| 1 | Slight increase in muscle tone, manifested by a catch and release or by minimal resistance at the end of motion when the affected part(s) in moved in flexion or extension |
| 1+ | Slight increase in muscle tone, manifested by a catch, followed by minimal resistance throughout the remainder (less than half) of the ROM |
| 2 | More marked increase in muscle tone through most of the ROM, but affected part(s) easily moved |
| 3 | Considerable increase in muscle tone, passive movement difficult |
| 4 | Affected part(s) rigid in flexion or extension |

1. OABSS (Day -28, 0, 3, 7, 14, 28)

The following questionnaire will be used to survey the period from the date of the last response to today.

| Question | Frequency | Score |
| --- | --- | --- |
| Q1. How many times do you typically urinate from walking in the morning to going to sleep at night? | 7 or less | 0 |
|  | 8-14 | 1 |
|  | 15 or more | 2 |
| Q2. How many times do you typically wake up to urinate at night? | None | 0 |
|  | 1 | 1 |
|  | 2 | 2 |
|  | 3 or more | 3 |
| Q3. How often do you have a sudden desire to urinate that is difficult to defer? | None | 0 |
|  | Less than once a week | 1 |
|  | Once a week or more | 2 |
|  | About once a day | 3 |
|  | 2-4 times a day | 4 |
|  | 5 times a day or more | 5 |
| Q4. How often do you leak urine because you cannot defer the sudden desire to urinate? | None | 0 |
|  | Less than once a week | 1 |
|  | Once a week or more | 2 |
|  | About once a day | 3 |
|  | 2-4 times a day | 4 |
|  | 5 times a day or more | 5 |

1. Assessment of pain and numbness using VAS (Day -28, 0, 3, 7, 14, 28)

For the period from the time of the last evaluation to today, ask the patient to write the severity of the problem on a line 10 cm long. (0 at the left end: "No pain (numbness)", 10 at the right end: "Pain (numbness) is at its worst")

# **Assessment of Safety**

## **Safety evaluation indicators**

1. Hematological tests: CBC
2. Biochemical serum tests: TP, AST, ALT, BUN, Cr, Na, K, FBS, CRP
3. Urinalysis: urine protein, urine sugar, occult blood, ketone body, urobilinogen, specific gravity, pH
4. Subjective findings (described in CTCAE v4.0-JCOG)

- General or systemic conditions at the time of administration
- Fever
- Skin and subcutaneous tissue disorder
- Gastrointestinal disorders: diarrhea, nausea, vomiting
- Metabolic and nutritional disorders: anorexia
- Nervous system disorders: somnolence

## **Assessment of Adverse Events**

For items (1) to (3) above (hematological tests, blood biochemical tests, and urinalysis), adverse events that fall under the following categories will be counted in CTCAEv4.0-JCOG.

- Grade 4 adverse events
- Grade 1/2/3 adverse events requiring hospitalization or prolonged hospitalization for treatment of the adverse event
- Grade 1/2/3 adverse events that are considered medically significant.

## **Recording Adverse Events**

The evaluation of safety assessment indicators in above will be conducted on Day 0, Day 7, Day 14, and Day 28, and recorded in the medical record and case report form. If the sub-investigator obtains safety information, he or she will record all safety information in the medical record and analyze the safety issues with the principal investigator. In the case of a serious event, a report on safety information will be submitted to the Kagoshima University Clinical Research Review Committee and the Director of the Kagoshima University Hospital.

## **Treatment and Follow-Up of Adverse Events**

Observation period of the subject of the clinical research after the occurrence of the disease, etc. Necessary examination and treatment will be taken as appropriate. If there is no improvement at the end of the study (21 days after the end of dosing; Day 28), evaluation and treatment will be continued until complete remission after the end of the study.

# **Statistics**

## **Data analysis**

Statistical analyst:

Eiji Matsuura, Associate Professor, Department of Neurology

Target number of targets and rationale for setting them:

Number of cases; 20 cases

Rationale for setting:

A pilot study (safety and efficacy confirmation study).

## **Statistical Analysis Plan**

The significance level for the entire study is set at 10% on one side. p-values are valid to the fourth decimal place. Unless otherwise noted, categorical data will be summarized in terms of frequencies and proportions, and continuous data will be summarized in terms of basic statistics. Basic statistics will be calculated as mean, standard deviation, minimum and maximum values. No interim analysis will be performed. Missing data will not be supplemented, and rejected data will be excluded. Regarding the handling of abnormal data, no judgment will be made as to whether the data is abnormal or not, and all data will not be excluded.

If there are any changes from the original statistical analysis plan, the research protocol will be revised and the changes will be explained in the summary report of the clinical research.

Discontinuation cases and dropout cases will be excluded from the analysis. However, the results up to the time of hospital discharge will be used for cases of post-discharge dropout (e.g., those who were not able to return to Japan for a follow-up visit) where medication has been completed and no adverse events have been observed in the discharge examination.

# **Access to Source Data/Documents**

The principal investigator and the implementing medical institution shall make all clinical research-related records, including source documents, available for direct inspection at the request of monitors, auditors, the Kagoshima University Clinical Research Review Committee, and regulatory authorities related to this clinical research.

# **Quality control and Quality Assurance**

## **Monitoring**

Monitoring will be conducted for some of the cases in this study. The purpose of this is to check the reliability of the data collected, compliance with the research protocol, and verification with the original documents. For this purpose, the person in charge will review the records (data, questionnaires, etc.). In addition, after the completion of the study, a third party who is not directly involved in the study will conduct an audit of the implementation system and procedures, and verification with the original materials.

(1) Implementation system: Kumiko Michizono, Project Assistant Professor, Center for Community Health Care Collaboration

(2) Implementation procedure: Once during the implementation year, it will be confirmed that personal information is appropriately handled and stored, and that the study is being conducted according to the protocol. The date and time of monitoring, the name of the monitor, the name of the physician in charge of explanation, the instructions given to the physician in charge, and the opinion of the monitor will be summarized, stored, and submitted to the principal investigator.

The principal investigator shall prepare a monitoring procedure for each research protocol, and conduct monitoring in accordance with said procedure and the research protocol.

## **Audit**

1. Head of Audit; Keiko Higashi, Project Assistant Professor, Regional Medical Support Center Implementation procedure: Once during the implementation year, the appointed person in charge of auditing will check the CRB approval document for this study, the subject consent document, and the case report data and medical records.
2. Implementation procedure: Once during the implementation year, for all cases, the appointed Audit Manager will check the CRB approval document for the study, check the subject consent document, and check the case report data against the medical records (direct access to the original documents). The audit will be conducted in accordance with the audit plan planned and submitted by the person in charge of audit, and the person in charge of audit will report and certify whether the subject of audit has been handled appropriately. Any matters pointed out in the audit will be reported and certified again by the person in charge of the audit after going through the designated documentation procedures.

Table 1: Schedule of Assessments

|  | Screening | Admission | Administration Drug | | | Discharge | Last visit |
| --- | --- | --- | --- | --- | --- | --- | --- |
| Permissible days | ±7 | －7 |  |  |  |  | ±7 |
| Hospital |  |  |  |  |  |  |  |
| Administration |  |  |  |  |  |  |  |
| Inclusion | ○ |  |  |  |  |  |  |
| Agreement | ○ |  |  |  |  |  |  |
| Back Ground^1)^ | ○ |  |  |  |  |  |  |
| Vital Signs | ○ | ○ | ○ | | | ○ | ○ |
| Height | ○ |  |  |  |  |  |  |
| Body Weight | ○ | ○ | ○ | ○ | ○ | ○ | ○ |
| CBC^2)^ | ○ | ○ | ○ | ○ | ○ | ○ | ○ |
| Blood Chemical^3)^ | ○ | ○ | ○ | ○ | ○ | ○ | ○ |
| Urination^4)^ | ○ | ○ | ○ | ○ | ○ | ○ | ○ |
| Blood Gas | When needed | When needed | When needed | When needed | When needed | When needed | When needed |
| Drug Concentration |  | ○ | ○ | ○ | ○ | ○ | ○ |
| Blood test^5)^ |  | ○ |  |  |  |  | ○ |
| CSF Study^6)^ |  | ○ |  |  |  |  | ○ |
| 10-meter walk test | ○ | ○ | ○ | ○ | ○ | ○ | ○ |
| Timed Up and Go Test | ○ | ○ | ○ | ○ | ○ | ○ | ○ |
| OMDS | ○ | ○ | ○ | ○ | ○ | ○ | ○ |
| General Findings Knee Clonus  MAS | ○ | ○ | ○ | ○ | ○ | ○ | ○ |
| OABSS | ○ | ○ | ○ | ○ | ○ | ○ | ○ |
| Pain/Numbness VAS | ○ | ○ | ○ | ○ | ○ | ○ | ○ |

○, Required

- 1. Initials, gender, date of birth, date of first diagnosis, medical history, treatment history, previous illness, complications, concomitant medications
  2. WBC, RBC, Hb, PLT, blood sedimentation rate
  3. TP, AST, ALT, BUN, Cr, Na, K, FBS, CRP, except Day-28 only, HbA1c
  4. Urine protein, urine glucose, occult blood, ketone body, urobilinogen, specific gravity, pH
  5. Serum sIL-2R, HTLV-1 proviral load in peripheral blood PBMC
  6. Cell count, protein, anti-HTLV-1 antibody titer (CLIA method), CSF neopterin concentration, CSF CXCL10
