# Supplementary material for: Efficacy of l‐Arginine treatment in patients with HTLV‐1‐associated neurological disease
Source: Ann Clin Transl Neurol. 2022 Dec 22;10(2):237–45. doi: 10.1002/acn3.51715 (PMC9930431; doi:10.1002/acn3.51715)
Supplement: Supplementary file 2 — Figure S1 [file ACN3-10-237-s002.docx]

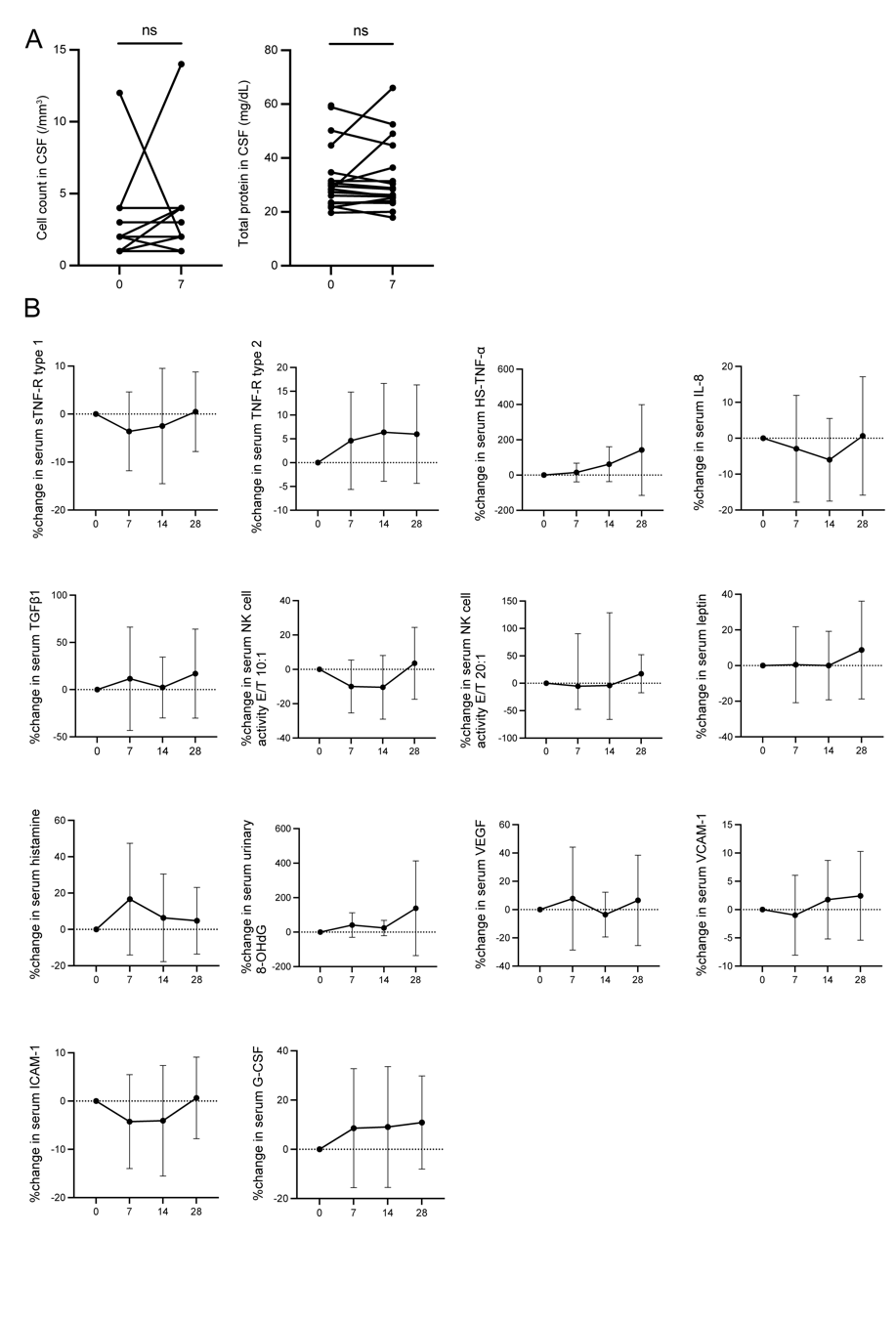


**Supplementary Figure 1. Change in immunological markers in the cerebrospinal fluid and peripheral blood of HAM/TSP patients treated with l-arginine.**

(A) Total cell counts (left) and total protein content (right) in the cerebrospinal fluid (CSF). (B) Percent change from baseline in serum levels of the indicated inflammatory biomarkers. sTNF-R, soluble tumor necrosis factor receptor; HS-TNF, high sensitivity tumor necrosis factor; IL, interleukin; TGF, transforming growth factor; NK, natural killer; E/T, effector to target ratio; 8-OHdG, 8-hydroxy-2′-deoxyguanosine; VEGF, vascular endothelial growth factor; VCAM-1, vascular cell adhesion molecule 1; ICAM-1, intercellular adhesion molecule 1; G-CSF, granulocyte colony stimulating factor. Dashed bars indicate baseline values. N=20. mean and 95% confidence interval.
