# Supplementary material for: Efficacy of l‐Arginine treatment in patients with HTLV‐1‐associated neurological disease
Source: Ann Clin Transl Neurol. 2022 Dec 22;10(2):237–45. doi: 10.1002/acn3.51715 (PMC9930431; doi:10.1002/acn3.51715)
Supplement: Supplementary file 3 — Figure S2 [file ACN3-10-237-s003.docx]

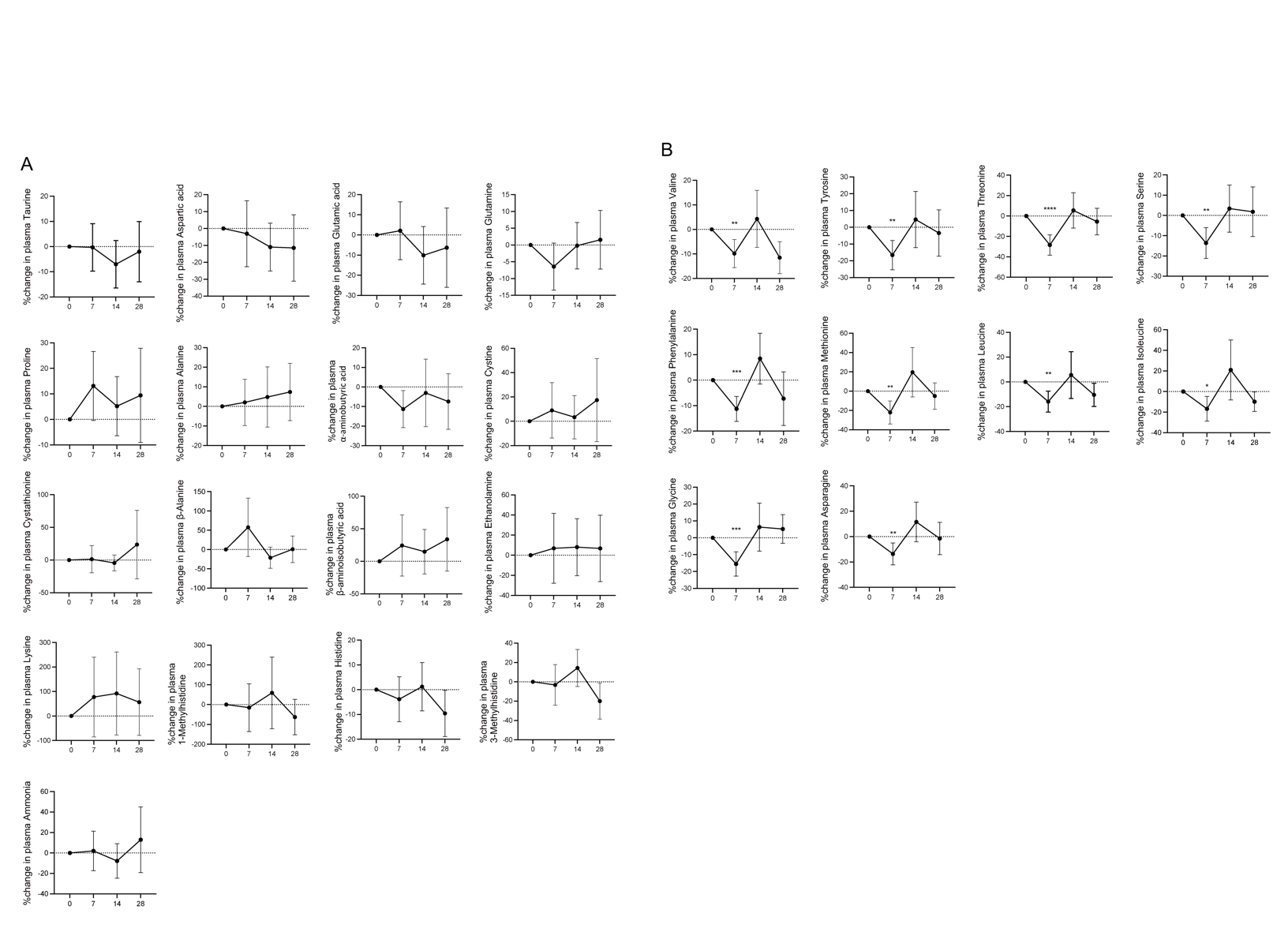


**Supplementary Figure 2. Percent change from baseline in plasma amino acid concentrations in HAM/TSP patients treated with l-arginine.**

(A) Amino acids exhibiting no significant change in plasma concentration. (B) Amino acids with significant decreases in plasma concentration. Dashed bar indicates baseline values. N=20. Mean and 95% confidence interval. **P* < .05, ***P* < .01, ****P* < .001, *****P* < .0001.
